# Acute kidney injury after liver resection in elderly patients

**DOI:** 10.1186/s12882-019-1449-0

**Published:** 2019-07-18

**Authors:** Ivana Dedinská, Peter Mikolajčík, Patra Skálová, Marián Mokáň, Ľudovít Laca

**Affiliations:** 1 Department of Surgery and Transplant Center, University Hospital Martin and Jessenius Medical Faculty, Comenius University, Kollárova 2, Martin, 036 01 Slovak Republic; 2 Ist Department of Internal Diseases, University Hospital Martin and Jessenius Medical Faculty, Comenius University Kollárova 2, Martin, 036 01 Slovak Republic

**Keywords:** Acute kidney injury, Liver resection, Geriatric patients

## Abstract

**Background:**

Acute kidney injury (AKI) affects approximately 13% of patients undergoing major abdominal surgery, and is a common and important clinical sign of perioperative injury. The aim of our analysis was to identify risk factors for AKI in elderly patients with no known kidney disease at the time of surgery, and to evaluate their 30-day, 12-month and 5-year survival.

**Methods:**

We performed a retrospective analysis on a group of 785 patients after liver resection to determine the incidence of complications (AKI – according to KDIGO classification, sepsis, cardiovascular and surgical complications). All patients had normal kidney function prior to surgery. We determined risk factors for the development of AKI for two groups of patients, stratified for age: patients younger than 65 years, and patients older than 65 years.

**Results:**

The incidence of complications was significantly higher in the group of patients older than 65 years (*n* = 76) than in younger patients (*n* = 119) (*P* = 0.0496). In the group of younger patients, significantly worse 30-day survival was observed for patients who developed AKI (*P* = 0.0004). We identified the following independent risk factors for AKI: male gender (HR 10,3834; *P* = 0,0238), histological identification of colorectal carcinoma metastases (HR 2,8651; *P* = 0,0499), surgery duration longer than 300 min (HR 6,0096; *P* < 0,0001), blood loss of more than 500 ml (HR 10,5857; *P* = 0,0012), and the need for more than 500 ml of fresh frozen plasma during surgery ml (HR 2,4878; *P* < 0,0317). Age was not confirmed to be an independent risk factor for AKI in our study.

**Conclusion:**

Approaches to treatment should be highly individualized, with assessment of several variables. According to our findings, age should not present a contraindication for the indication of a patient for surgery.

## Background

Acute kidney injury (AKI) is a global public health concern, associated with high morbidity, mortality (approximately 1.7 million deaths per year) and healthcare costs [1]. The systemic inflammatory response to infection, trauma and surgery are the most important moments for AKI. Systemic inflammation is already well known to cause stress or injury to endothelial, tubular and glomerular kidney cells which are very sensitive to circulating inflammatory. Systemic inflammation stimulates adaptive responses in several kidney cells (glomerular and tubular). The clinical outcomes of such responses depend on the stage of inflammatory stress, and may range from mild proteinuria to glomerular filtration rate (GFR) loss, requiring renal replacement therapy and lead to significantly increase of short- and long-term mortality risk [2].

Convincing evidence suggests that the incidence of AKI is rapidly increasing, especially among acutely ill hospitalized patients and patients undergoing major surgical procedures. This increase can partly be thanks to greater recognition of AKI, improved detection of administrative data and greater sensitivity of diagnostics and classification schemes. Other causes include an aging population and increases in the prevalence of cardiovascular disease, diabetes mellitus and chronic kidney disease [3]. Elderly people have aging kidneys that are undergoing structural and functional changes, connected with decreased autoregulatory capacity and increased susceptibility to damage [4]. The incidence rate of AKI is higher among the elderly population than younger populations, and age is recognized to be a major predictive factor for mortality in patients with AKI [5]. Geriatric patients are typically graded into the following age groups: young-old age (65–74 years), middle-old age (75–84 years), and old-old age (85 years and older) [6, 7].

Within the scope of cancer treatment, liver resection is the treatment of choice for many primary and secondary diseases of the liver. Most studies of elderly patients undergoing this procedure report the resection of primary and secondary liver tumors, especially hepatocellular carcinoma and colorectal metastatic cancer [8]. However, hepatectomy has become more common over the past two decades, and is also performed in the older population, demonstrating a paradigm shift in the treatment approach for these patients [9].

Acute kidney injury affects around 13% of patients undergoing major abdominal surgery. On average, AKI is associated with a 12-fold (95% CI [6.8, 23.4]) increase in the crude risk of death during the postoperative period. As AKI is a common and important clinical sign of perioperative harm, it represents a potential target for measures to improve postoperative outcomes [10].

There is currently no data in the literature on AKI after liver resection in elderly patients. The aim of our analysis was to identify risk factors for AKI in elderly patients with no known kidney disease at the time of surgery, and to evaluate their 30-day, 12-month and 5-year survival.

## Methods

**Fig. 1:**
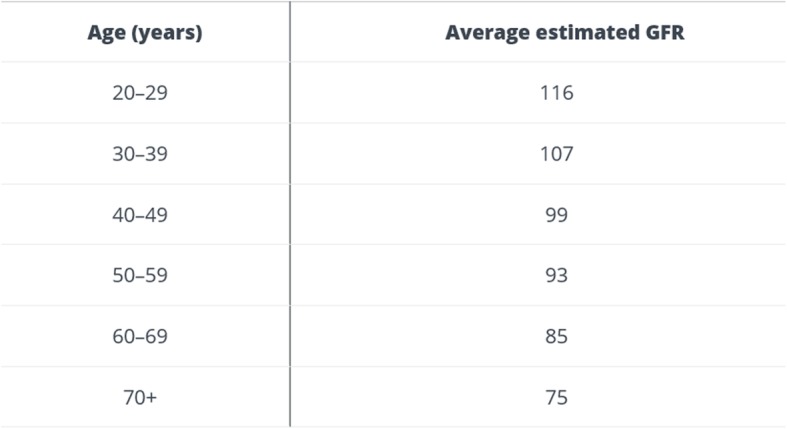
Average estimated GFR (ml/min) based on age

We performed a retrospective analysis of a group of patients who underwent liver resection between June 2003 and March 2018 in University hospital Martin. Patients with known kidney disease or diabetes mellitus, and those with poor glomerular filtration (eGFR according to CKD-EPI) for their age, classified according to the National Kidney Foundation (Fig. 1) [11], were excluded from monitoring. Patients with reduced eGFR were excluded from the group with regard to the effort to create a homogeneous group of patients with normal kidney function. It is generally known that patients with compromised renal function have increased AKI risk and we tried to identify in our analysis risk patients for AKI in the input “non-risk” group of patients without reduced eGFR at the time of surgery.

We identified the cause of resection (according to histological findings) and the type of resection performed, classified as either large resection (hemihepatectomy or extended hemihepatectomy), small resection (resection of segments), or radiofrequency ablation (RFA; performed when the liver could not be resected).

We divided the patients into two groups according to their age at the time of the operation (younger than 65 years and older than 65 years) according to WHO classification of seniors [12]. For each patient, we recorded the presence of postoperative complications (cardiovascular, septic, surgical or AKI) and their relationship to the type of resection and patient age. Cardiovascular complications included cardiac failure after surgery, arrhythmia and acute coronary syndrome. Sepsis was defined according to following criteria:

(1) sepsis, systematic inflammatory reaction characterized by the presence of a minimum of two of (a) body temperature over 38 °C or below 36 °C, (b) heart rate exceeding 90 beats/min (c) respiratory rate exceeding 20 breaths/min or hyperventilation with decreased PaCO_2_ under 4.3 kPa, and (d) abnormal number of white blood cells (over 12,000/mm^3^ or under 4000/mm^3^) or the presence of more than 10% of immature forms of leukocytes; (2) severe sepsis, the transition between sepsis and septic shock characterized by the presence of at least one of the following symptoms of organ hypoperfusion of (a) qualitative or quantitative consciousness disorder, (b) hypoxemia characterized by PaO_2_ under 10 kPa, (c) lactatemia over 2.5 mmol/L, and (d) oliguria under 30 ml/h or under 0.5 ml/kg/h; (3) septic shock, characterized by symptoms of sepsis or severe sepsis in addition to hypotension insufficient to complete the volume of liquids and signs of organ hypoperfusion [13].

Surgery complications were defined as the presence biliary leakage and/or the presence of bleeding, either requiring reoperation or treated by the administration of blood derivatives and hemostyptic treatment.

Postoperative AKI (up to 30 days after the surgery) was defined according to the KDIGO (Kidney Disease Improving Global Outcomes) classification.

Postoperative complications were defined as a complication which developed up to 30 days after the surgery. We also determined the 30-day survival of patients in both age groups and according to the type of complication developed by the patient during the monitored period.

We used a certified statistical program, MedCalc version 13.1.2. (VAT registration no. BE 0809 344 640, Member of International Association of Statistical Computing, Ostend, Belgium), to perform statistical analyses. Parametric (t-test) or non-parametric (Mann–Whitney) tests were used for comparisons of continuous variables and the χ2 test and Fisher’s exact test were used for categorical variables, as appropriate. Cox proportional hazard model was used for multivariate analysis (AKI development as the outcome variable and monitored parameters as the covariates) and Kaplan-Meier curves were used for survival analyses. We considered a *P*-value of < 0.05 to be statistically significant.

## Results

The study group included 785 patients (423 males and 362 females) with an average age of 58.7 ± 11.7 years (range 20–80 years) at the time of surgery. The group of patients aged younger than 65 years consisted of 510 patients (65%) and the group of patients older than 65 years consisted of 275 patients (35%). We identified 195 patients (25%) who developed postoperative complications during the monitored period. We recorded postoperative complications in 119 patients (23.3%) in the group younger than 65 years, and in 76 patients (27.6%) in the group older than 65 years (*P* = 0.0496). Characteristics of the group are presented in Table 1. Cardiologic complications occurred significantly more often in the group of older patients, whereas sepsis and surgical complications were more frequent in the group of younger patients. Younger patients underwent large resection of liver and RFA significantly more often than older patients. Regarding the histological findings, benign findings and neuroendocrine tumors were more common in younger patients, whereas colorectal carcinoma with metastases in the liver were more frequent in the group of patients older than 65 years. We recorded greater perioperative blood loss in patients older than 65 years.

**Table 1:** Group characteristics and patient demographics

	< 65 years (*n* = 119)	≥65 years (*n* = 76)	*P-value*
Cardiologic complications	15 (12.6%)	30 (39.5%)	**< 0.0001**
Sepsis	41 (34.5%)	17 (22.4%)	**0.0132**
Surgical complications	47 (39.5%)	19 (25%)	**0.0031**
AKI (KDIGO)	16 (13.4%)	10 (13.2%)	0.8895
Small resection	16 (13.4%)	10 (13.2%)	0.9325
Large resection	59 (49.6%)	48 (63.2%)	**0.0172**
RFA	44 (40%)	18 (23.7%)	**0.0091**
Benign finding	24 (20.2%)	8 (10.5%)	**0.0123**
HCC	8 (6.7%)	8 (10.5%)	0.1811
Cholangiocarcinoma	16 (13.4%)	11 (14.5%)	0.7797
MTS of colorectal carcinoma	29 (24.4%)	35 (46%)	**< 0.0001**
Neuroendocrine tumors	10 (8.4%)	3 (3.9%)	**0.0333**
Other	32 (26.9%)	11 (14.5%)	**0.0062**
Duration of surgery (min)	216 ± 100	228 ± 99	0.2453
Perioperative blood loss (ml)	451 ± 425	623 ± 591	**0.0009**
Substitution of FFP (ml)	443 ± 414	472 ± 399	0.4930

*AKI*, acute kidney injury, *RFA* radiofrequency ablation, *HCC* hepatocellular carcinoma, *MTS* metastases, *ČMP* fresh frozen plasma

**Fig. 2:**
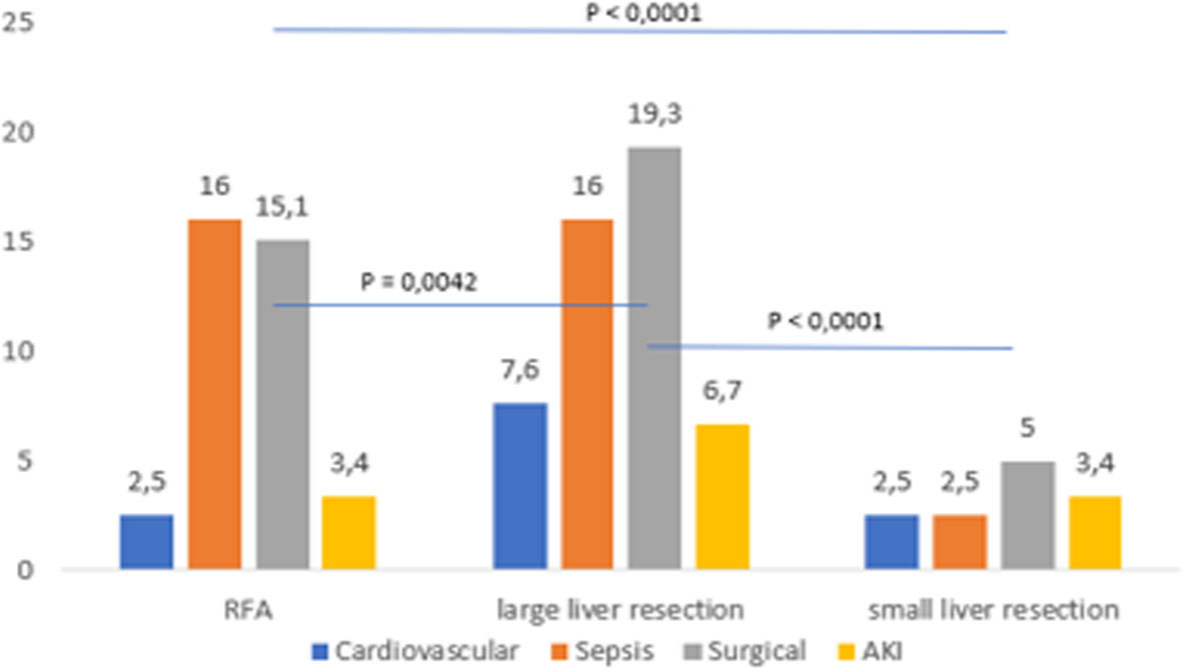
Complications (%) among patients aged younger than 65 years according to the type of surgery

**Fig. 3:**
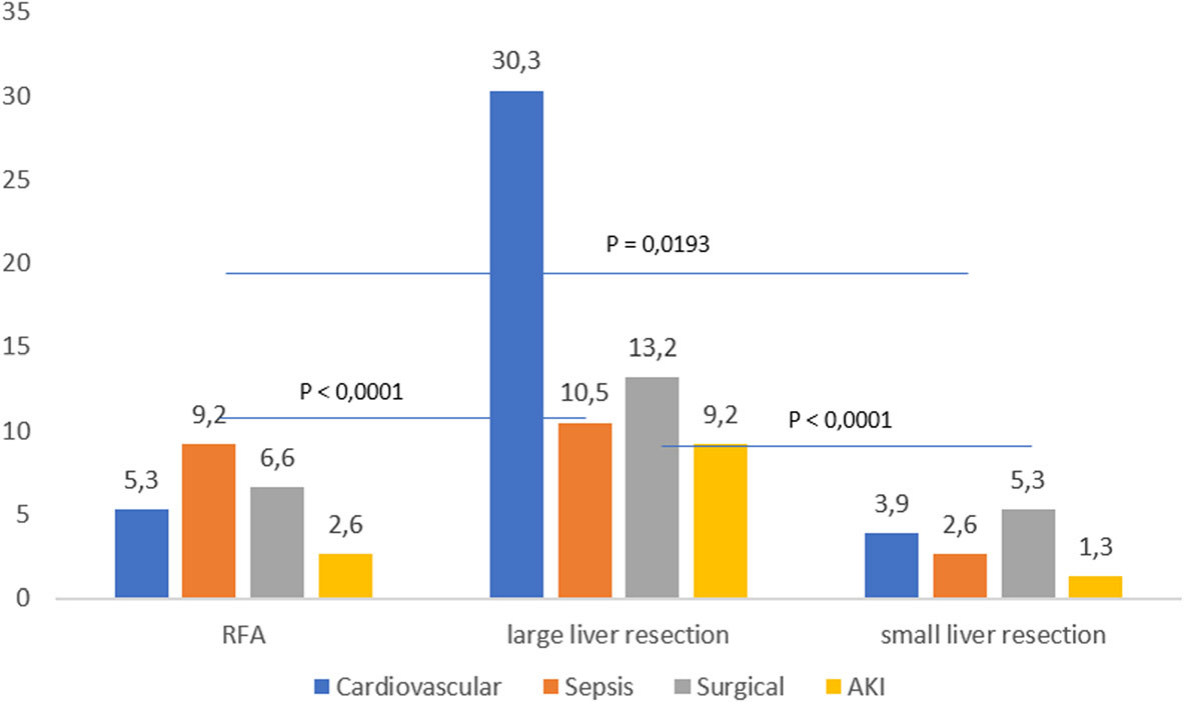
Complications (%) among patients older than 65 years according to the type of surgery

Figures 2 and 3 show individual complications according to the type of surgery. We recorded significantly fewer complications from small liver resection procedures in both groups.

**Fig. 4:**
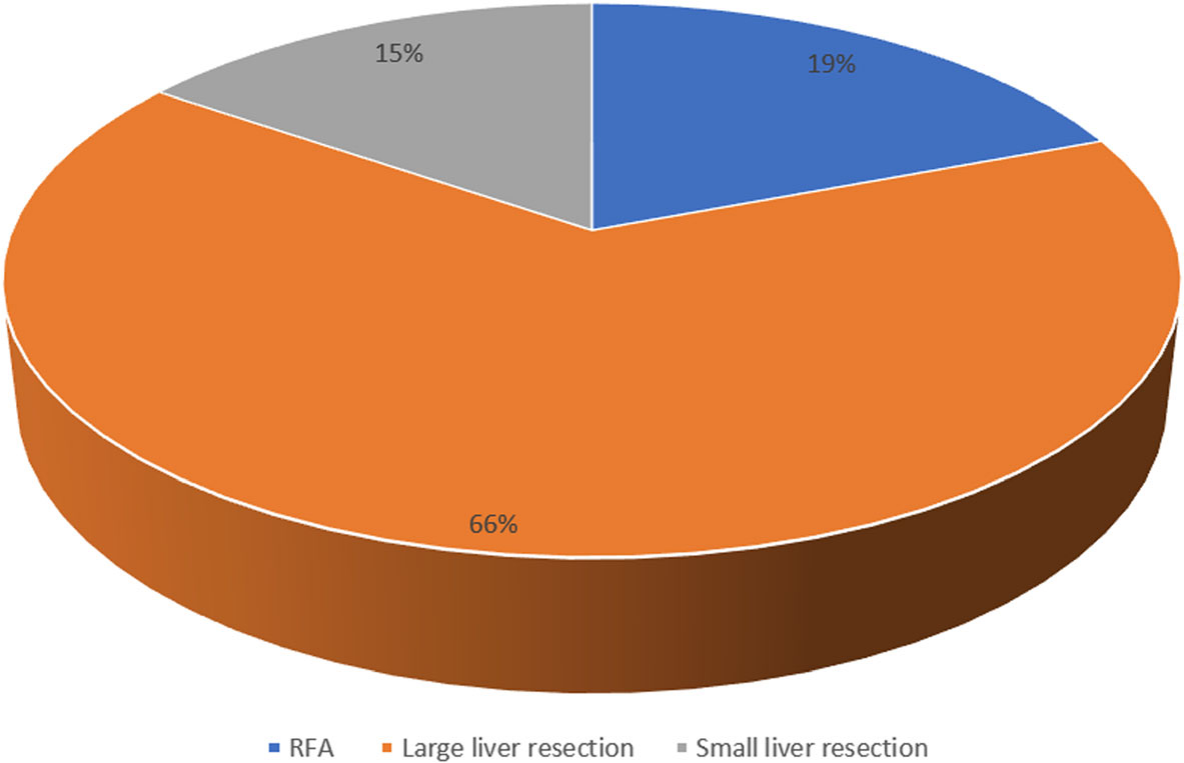
Distribution of the group who developed acute kidney injury (AKI) according to the type of surgery

Of the 195 patients with complication development in 30 day after surgery follow up, 26 patients developed AKI. We found that patients younger than 65 years who developed AKI had significantly better preoperative eGFR in comparison with older patients with AKI development, *P* < 0.0001 (Table 2). Group characteristics of patients with AKI (Table 3). AKI KDIGO stage 1 was more common in patients with AKI (in both younger and older patients), followed by the development of AKI KDIGO stage 2 diagnosed in 23.8% of younger patients and 17.6% of older patients, with no significant difference between the groups. AKI KDIGO stage 3 was diagnosed in 23.8% of younger patients and 8.8% of older patients. Acute kidney injury occurred more regularly after large liver resection, and AKI KDIGO 3 occurred only in patients who had undergone large liver resection. Distribution of patients with AKI according to the type of surgery is shown in Fig. 4.

**Table 2:** Base line characteristics (before surgery) – patients who developed AKI

	< 65 years (*n* = 17)	≥65 years (*n* = 9)	*P-value*
Creatinine (μmol/L)	54.3 ± 12.4	69.7 ± 23.1	**0.0022**
eGFR (mL/min/1.73m^2^)	108 ± 10.8	80.4 ± 12.6	**< 0.0001**

**Table 3:** AKI group characteristics

	AKI (*n* = 26)
Age < 65 years	16
Age ≥ 65 years	10
Small resection	4
Large resection	17
RFA	5
Benign finding	5
HCC	1
Cholangiocarcinoma	2
MTS of colorectal carcinoma	16
Neuroendocrine tumors	1
Other	1
Duration of surgery (min)	224 ± 84,7
Perioperative blood loss (ml)	402.5 ± 124
Substitution of FFP (ml)	357.5 ± 283.6

*AKI* acute kidney injury, *RFA* radiofrequency ablation, *HCC* hepatocellular carcinoma, *MTS* metastases, *FFP* fresh blood plasma

Application of correlation coefficient did not show any statistically significance dependence on age and occurrence of AKI, both in the whole group (*r* = 0.1325; *P* = 0.2670) and in the group of younger patients (*r* = − 0.1084; *P* = 0.4488) or older patients (*r* = 0.3428; *P* = 0.2062). Application of probit regression on the whole group as well as on individual monitored groups (younger than 65 years, 65–74 years and more than 75 years) did not show any statistical significance between the age and probability of AKI occurrence.

**Fig. 5:**
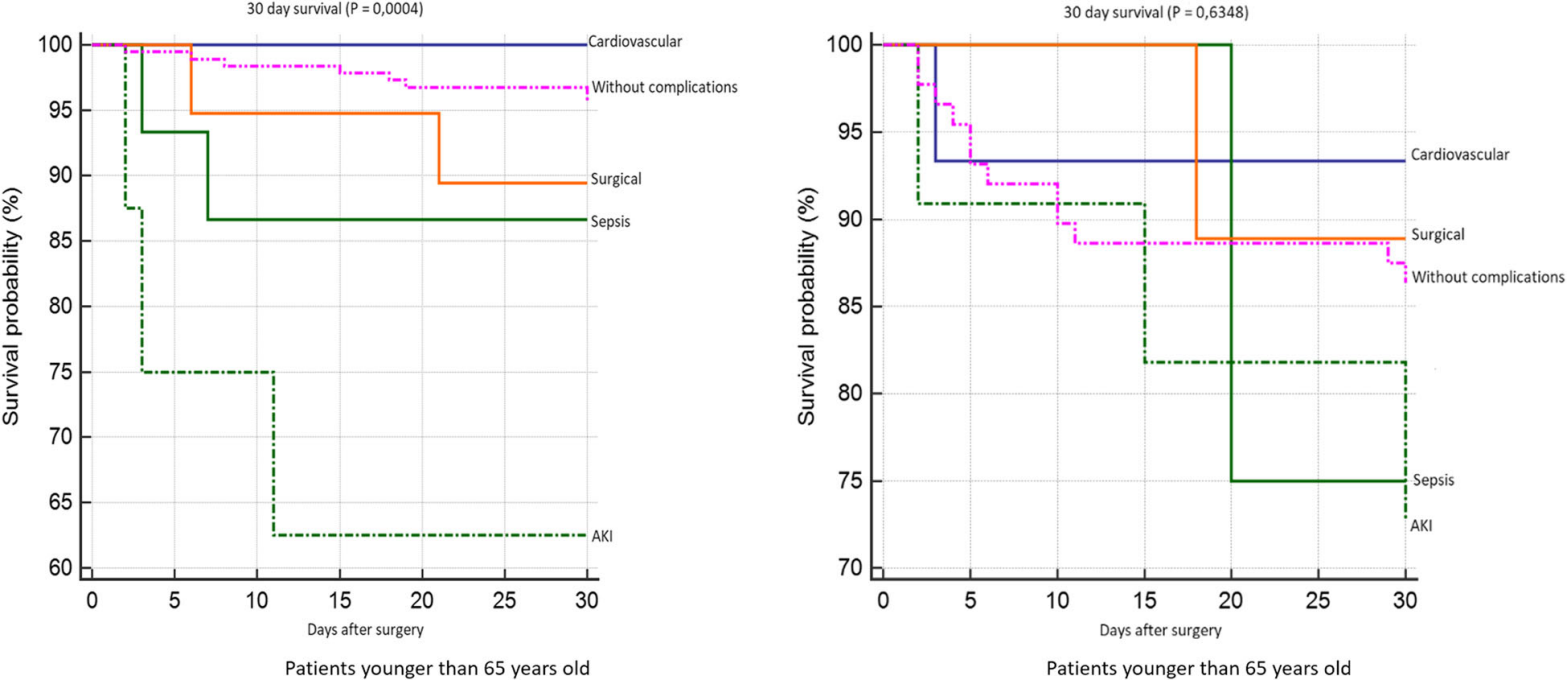
30-day survival according to individual complications

**Fig. 6:**
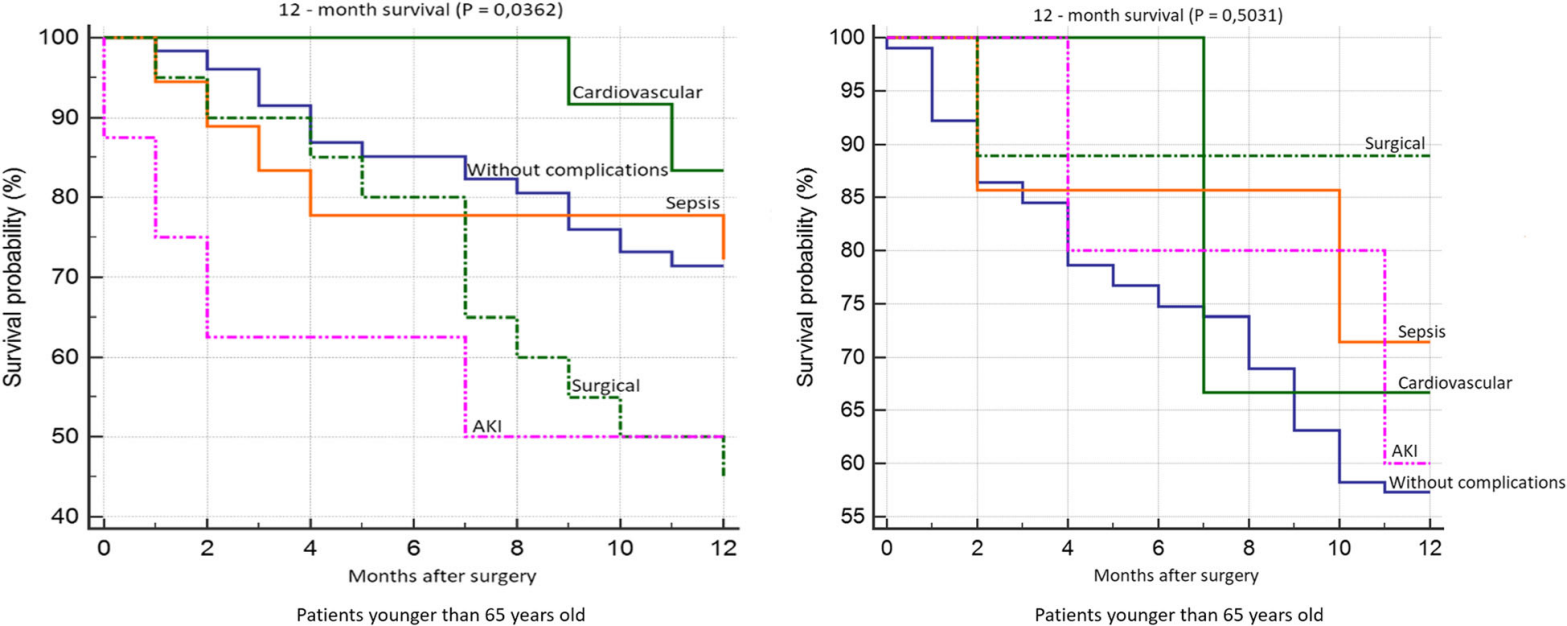
12 month survival according to individual complications

**Fig. 7:**
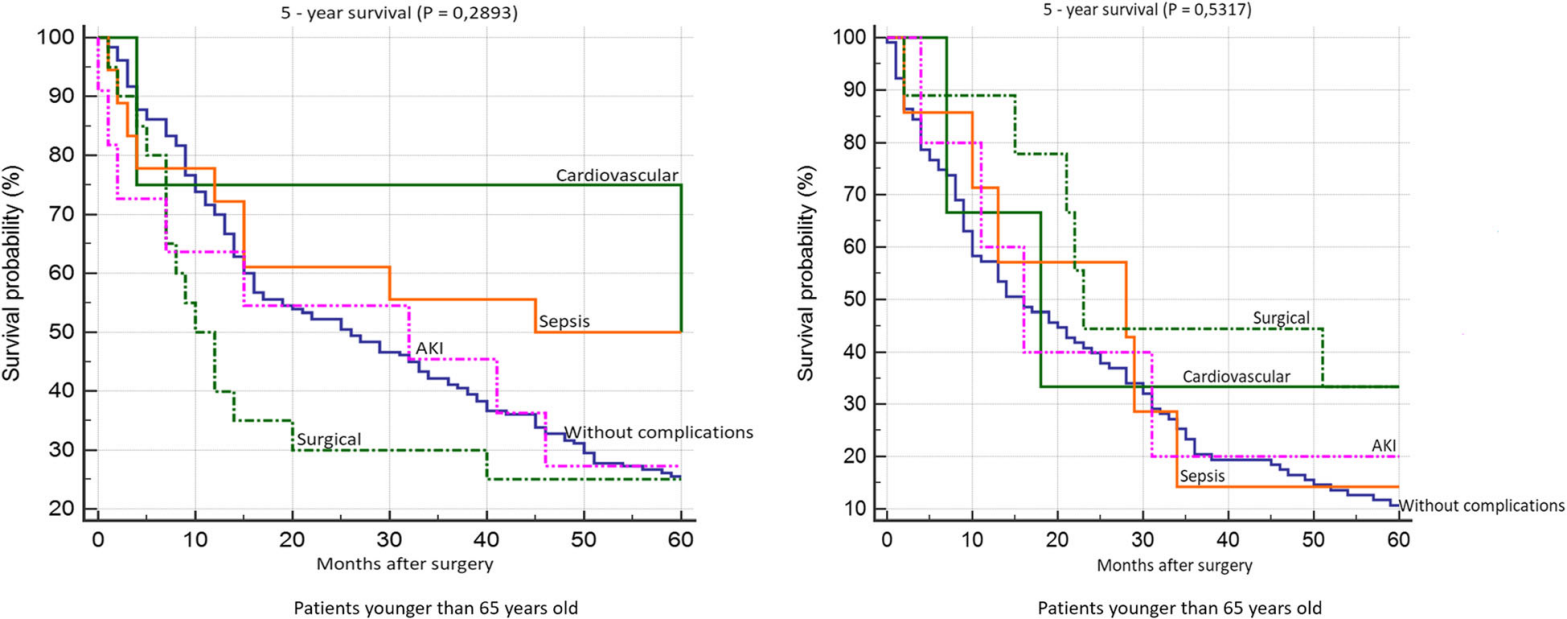
5 year survival according to individual complications

**Fig. 8:**
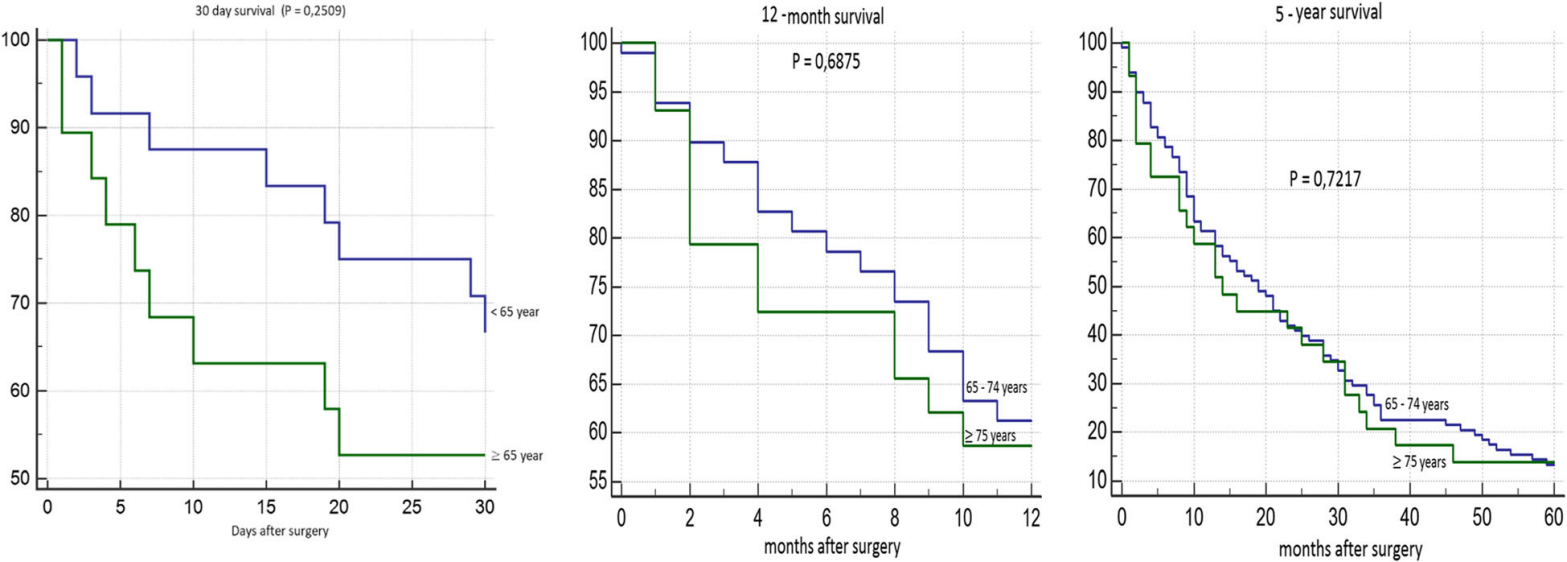
30-day, 12-month and 5 year survival of patients with acute kidney injury (AKI) according to age

Patient survival 30 days after surgery is shown in Fig. 5. We found significantly worse survival in the group of younger patients who developed AKI. Survival rates according to individual complications did not significantly differ in the group of older patients. 12-month survival of patients younger than 65 years was significantly worst in the group of patients with AKI and with surgical complications. We did not confirm any statistically significant difference in patient survival according to individual complications in the group of older patients (Fig. 6). We did not confirm any statistically significant difference in 5-year survival both in the group of older and younger patients (Fig. 7). Finally, we evaluated 30-day survival of patients with AKI by multivariate analysis, but no significant difference between older and younger patients was found. Finally we compared the survival of patients older than 65 years. We divided this group to patients older than 75 years and patients in age 65–74 years. We did not find any statistically significant difference in 12-month and 5-year survival (Fig. 8).

Using multivariate analysis, the following independent risk factors for AKI were identified: male gender, histological finding of colorectal carcinoma metastases, duration of surgery exceeding 300 min, blood loss greater than 500 ml, and the necessity for more than 500 ml of fresh frozen plasma during surgery (Table 4). Age was unproven to be an independent risk factor for AKI within our group.

**Table 4:** Cox proportion hazards model for the whole group

AKI	Hazard ratio	95% CI	*P-value*
Gender (male)	10.3834	1.3644, 79.0199	**0.0238**
Cardiologic complications	3.5510	0.1925, 65.4912	0.3941
Sepsis	0.4595	0.02807, 7.5219	0.5856
Surgical complications	1.7433	0.4030, 7.5418	0.4570
Age < 65 years	0.8721	0.2914, 2.6102	0.8067
Age 65–74 years	1.0346	0,2913, 3,6659	0.0507
Age ≥ 75 years	0.4418	0,02697, 7,2381	0.5669
Small resection	1.1540	0.2637, 5.0504	0.8492
Large resection	2.0226	0.6747, 6.0632	0.2085
RFA	0.5974	0.1351, 2.6421	0.4970
Benign finding	0.9467	0.2155, 4.1598	0.9422
HCC	0.4361	0.02803, 6.7845	0.5534
Cholangiocarcinoma	1.0705	0.1448, 7.9150	0.0668
MTS of colorectal carcinoma	2.8651	0.9571, 8.5764	**0.0499**
Neuroendocrine tumors	1.7917	0.2480, 12.9440	0.5633
Other	0.2228	0.02935, 1.6909	0.1465
Duration of surgery > 300 min	6.0096	3.1117, 11.6063	**< 0.0001**
Perioperative blood loss > 500 ml	10.5857	2.5384, 44.1456	**0.0012**
Substitution of FFP > 500 ml	2.4878	0.9228, 6.7066	0.0717

*AKI* acute kidney injury, *RFA* radiofrequency ablation, *HCC* hepatocellular carcinoma, *MTS* metastases, *FBP* fresh frozen plasma

With regard to the results of the multivariate analysis, we further compared men (*n* = 101) and women (*n* = 190) who developed complications. We found that women developed sepsis significantly more often than men, which is probably related to the greater number of RFA procedures performed in this group. On the other hand, AKI was more often diagnosed in men, which may be related to the significantly higher number of large liver resections in men, associated with significantly higher blood loss and the need to use larger volumes of fresh frozen plasma. Furthermore, hepatocellular carcinoma was more frequent in men (Table 5).

**Table 5:** Comparison of complications between male and female patients

	Males (*n* = 101)	Females (*n* = 94)	*P-value*
Cardiologic complications	23 (22.8%)	22 (23.4%)	0.4723
Sepsis	17 (16.8%)	41 (43.6%)	**< 0.0001**
Surgical complications	37 (36.6%)	29 (30.9%)	0.2020
AKI	24 (25.2%)	2 (2.1%)	**< 0.0001**
Age < 65 years	59 (58.4%)	60 (63.8%)	0.3837
Age ≥ 65 years	42 (41.6%)	34 (36.2%)	0.3837
Small resection	6 (5.9%)	20 (21.3%)	**< 0.0001**
Large resection	73 (72.3%)	34 (36.2%)	**< 0.0001**
RFA	22 (21.8%)	40 (42.6%)	**< 0.0001**
Benign finding	14 (13.9%)	18 (19.1%)	0.2850
HCC	12 (11.9%)	4 (4.3%)	**0.0055**
Cholangiocarcinoma	12 (11.9%)	15 (16%)	0.2637
MTS of colorectal carcinoma	45 (44.6%)	19 (20,2%)	**< 0.0001**
Neuroendocrine tumors	7 (6.9%)	6 (6.4%)	0.8114
Other	11 (10.9%)	32 (34%)	**< 0.0001**
Duration of surgery (min)	227 ± 106	212 ± 92	0.1364
Perioperative blood loss (ml)	580 ± 532	460 ± 427	**0.0146**
Substitution of FFP (ml)	480 ± 473	390 ± 358	**0.0351**

*AKI* acute kidney injury, *RFA* radiofrequency ablation, *HCC* hepatocellular carcinoma, *MTS* metastases, *FFP* fresh blood plasma

## Discussion

Liver surgery is a major procedure which presents a challenge for anesthesiologists and surgeons, in addition to the patient [14]. The risk of perioperative complications depends on the condition of the patient prior to surgery, the presence of comorbidities, and the urgency, magnitude, type and duration of the surgical procedure [15]. As expected, a significantly higher incidence of cardiologic complications in older patients was observed. On the other hand, surgical complications were more frequent in younger patients, which were linked to the type of surgery, with a higher prevalence in those who underwent large liver resection. Septic complications are linked to RFA, which, despite being a relatively safe method, can lead to infection associated with necrotic tissue in the RFA site [16].

As for the histological findings, it was discovered that colorectal carcinoma with metastases in the liver was more common in older patients. It had been previously reported that more than 60% of patients with colorectal carcinoma were aged over 70 years [17]. Contrary to this, benign tumors and neuroendocrine tumors were more common in younger patients.

The occurrence of AKI after liver resection in the literature ranges from 0.9–15%. In the current study, AKI developed in 3.3% of patients. Low occurrence of AKI in our study is influenced by the selection of patients (exclusion of patients with reduced eGFR). It was assumed that age would be an important risk factor for the development of AKI, but this was unconfirmed in our analysis. Therefore, older patients with normal kidney function prior to surgery do not appear to have a higher risk of postoperative AKI. However, if AKI does develop, these patients have significantly worse 30-day postoperative survival when compared to younger patients. Korean authors indicated in their retrospective analysis with 228 patients and AKI incidence after partial liver resection 11.8% that patients with AKI after liver resection may be at higher risk of mortality or moderate renal dysfunction within 3 years [18].

Male gender was found to be an independent risk factor for AKI in our group, which led us to conduct an additional analysis considering gender. Large liver resection with higher blood loss and the need for.

a larger volume of freshly frozen plasma was significantly more common in males. These factors are associated with a higher risk of postoperative AKI development. The authors of an earlier retrospective analysis of 446 patients also identified severe liver resection and perioperative hemodynamic instability as risk factors for AKI. Although these authors regarded chronic kidney disease as the most important factor in AKI, these patients were excluded from our analysis and therefore our findings are not affected by the patient’s disease [19].

Acute kidney injury most commonly occurred after severe liver resection and AKI stage 3 was only developed in patients undergoing severe liver resection. Large liver resection is also associated with longer duration of surgery, greater operative blood loss, hemodynamic instability with renal hypoperfusion, and subsequent development of AKI. Peres of al. In a review study on AKI after partial hepatectomy, blood loss by renal hypoperfusion during surgery is considered the most important risk factor for AKI, while the second factor is associated with liver failure after hepatectomy followed by distributional circulation changes and hepatorenal syndrome. The combination of individual triggers or the consumption of nephrotoxic drugs is considered an important factor [20]. Important measures for the prevention of postoperative AKI after partial hepatectomy would be appropriate preoperative treatment, careful patient selection for surgery and strict perioperative haemodynamic control of the patient [20].

To reduce the incidence of postoperative AKI after partial hepatectomy, careful patient selection and preoperative resection planning is required. Measures should be taken to prevent persistent intraoperative hypotension and postoperative bleeding, as well as the prevention and rapid treatment of sepsis. In situations of patients at high-risk for developing postoperative AKI, the nephrologist must promptly engage in multidisciplinary discussions in order to improve patient outcomes [19, 21].

The limitations of our study are as follows: a small, single-centre, retrospective dataset.

## Conclusions

Partial hepatectomy is the current treatment option for a variety of liver and biliary disorders. Among the potential complications of major surgery, including partial hepatectomy, acute renal impairment should be considered as an important cause of increased morbidity and postoperative mortality.This approach should be customized for each patient by evaluating multiple variables. According to our results in the group of risk patients with normal preoperative GFR, age should not be the only contraindication for the indication of surgery. A multidisciplinary approach during pre-hospitalization and during surgery may help to control the hemodynamic condition of the patient, which is critically important for AKI prevention.

## Data Availability

The data that support the findings of this study are available from University hospital Martin but restrictions apply to the availability of these data, which were used under license for the current study, and so are not publicly available. Data are however available from the authors upon reasonable request and with permission of University hospital Martin.

## Abbreviations

AKI: Acute kidney injury
eGFR: estimated glomerular filtration rate
KDIGO: Kidney Disease Improving Global Outcomes
RFA: Radiofrequency ablation

## References

[1] Mehta RL, Cerda J, Burdmann EA (2015). International Society of Nephrology's 0by25 initiative for acute kidney injury (zero preventable deaths by 2025): a human rights case for nephrology. Lancet.

[2] Mårtensson Johan, Bellomo Rinaldo (2017). Acute Kidney Injury. Inflammation - From Molecular and Cellular Mechanisms to the Clinic.

[3] Rewa O, Bagshaw SM (2014). Acute kidney injury—epidemiology, outcomes and economics. Nat Rev Nephrol.

[4] Anderson S, Eldadah B, Halter JB (2011). Acute kidney injury in older adults. J Am Soc Nephrol.

[5] Uchino S, Kellum JA, Bellomo R, et al. Acute renal failure in critically ill patients: a multinational, multicenter study. JAMA. 2005;294(7):813-8. 10.1001/jama.294.7.813 16106006

[6] Health and Welfare Canada. Health Promotion Survey. Ottawa: Health Welfare.

[7] Hegyi L, Krajčík S (2010). Geriatria. Herbena.

[8] Brunken C, Rogiers X, Malago M (1998). Is resection of colorectal liver metastases still justified in very elderly patients. Chirurg.

[9] Caratozzolo E, Massani M, Recordare A (2007). Liver resection in elderly: comparative study between younger and older than 70 years patients. Outcomes and implications for therapy. G Chir.

[10] O’Connor ME, Kirwan CJ, Pearse RM (2016). Incidence and associations of acute kidney injury after major abdominal surgery. Intensive Care Med.

[11] 2018 National Kidney Foundation, online. https://www.kidney.org/atoz/content/gfr.

[12] World Health Organisation. 10 facts on ageing and the life course. 2012. http://www.who.int/features/factfiles/ageing/ageing_facts/en/index.html. Accessed 2 Feb 2014.

[13] Singer M, Deutchman CS, Seymour CW (2016). The third international consensus definitions for Sepsis and septic shock (Sepsis-3). JAMA.

[14] Melloul E, Hübner M, Scott M (2016). Guidelines for perioperative care for liver surgery: enhanced recovery after surgery (ERAS) society recommendations. World J Surg.

[15] Kristensen SD, Knuuti J, Saraste A (2014). 2014 ESC/ESA guidelines on non-cardiac surgery: cardiovascular assessment and management: the joint task force on non-cardiac surgery: cardiovascular assessment and management of the European Society of Cardiology (ESC) and the European Society of Anaesthesiology (ESA). Eur Heart J.

[16] Razafindratsira T, Isambert M, Evrard S (2011). Complications of intraoperative radiofrequency ablation of liver metastases. HPB (Oxford).

[17] Millan M, Merino S, Caro A (2015). Treatment of colorectal cancer in the elderly. World J Gastrointest Oncol.

[18] Ishikawa S, Tanaka M, Maruyama F (2017). Effects of acute kidney injury after liver resection on long-term outcomes. Korean J Anesthesiol.

[19] Bredt LC, Peres AB (2017). Risk factors for acute kidney injury after partial hepatectomy. World J Hepatol.

[20] Peres AB, Bredt LC, Cipriani RFF (2016). Acute renal injury after partial hepatectomy. World J Hepatol.

[21] KDIGO Clinical Practice Guideline for Acute Kidney Injury (2012). Kidney Int.

